# Cannabis Use versus Cigarette Smoking in Emergency Department Patients: Comparative Outcomes

**DOI:** 10.5811/westjem.64353

**Published:** 2026-10-14

**Authors:** Vincent Yang, Alexander V. Xavier, Karthik Devulapally, Krishna K. Paul, Bryan Wilson, Diann Gaalema, Hani Jneid, Olugbenga Akinwande, M. Kristen Peek, Dietrich von Kuenssberg Jehle

**Affiliations:** * University of Texas Medical Branch at Galveston, Department of Emergency Medicine, Galveston, Texas; † University of Texas Medical Branch at Galveston, Department of Cardiovascular Medicine Galveston, Texas; ‡ University of Texas Medical Branch at Galveston, Department of Population Health & Health Disparities, Galveston, Texas

## Abstract

**Introduction:**

Cannabis is one of the most commonly used psychoactive substances worldwide, with 20% of adults in the U.S. reporting using cannabis at least once in the past year. Nicotine dependence is reported in 9% to 10% of adults in the U.S.^1^ This study aims to investigate the relationship between cannabis use and cigarette smoking on mortality, cardiovascular outcomes, suicide, cancer of the lung and bronchus, and reactive airway disease in emergency department (ED) patients.

**Methods:**

This retrospective, propensity score–matched study used the TriNetX database to analyze records of 120,689,106 patients from 69 healthcare organizations in the U.S. between 2005 and 2022. The first cohort consisted of adult patients and was grouped based on diagnosis of cannabis-related disorders (CRD) within five years before or one month after an ED visit. The second cohort consisted of adult patients with a diagnosis of “nicotine dependence, cigarettes” within five years before to one month after an ED visit. The outcomes evaluated were all-cause mortality, stroke, myocardial infarction, suicide, cancer of the lung and bronchus, and reactive airway disease (asthma/chronic obstructive pulmonary disease [COPD]) within three years after the ED visit. Patients who previously experienced these outcomes or belonged to both cohorts were excluded from the study. We performed 1:1 greedy nearest-neighbor propensity score matching for demographics and seven preexisting conditions associated with mortality.

**Results:**

There were 1,682,117 patients identified with ED visits and CRD or cigarette smoking. After propensity score matching, there were a total of 651,232 adult patients with CRD (*n* = 325,616) or cigarette smoking (*n* = 325,616) within five years prior or one month after an ED visit. Patients with CRD were associated with a significantly higher mortality rate (2.35% vs 2.16%; relative risk [RR] 1.09; 95% CI, 1.05–1.12; *P* < .001), stroke (0.45% vs 0.28%; RR 1.59; 95% CI, 1.47–1.73; *P* < .001), myocardial infarction (0.53% vs 0.35%; RR 1.51; 95% CI, 1.40–1.63; *P* < .001) and suicide (0.62% vs 0.35%; RR 1.77; 95% CI, 1.65–1.90; *P* < .001) within 3-years of the ED visit compared to patients smoking cigarettes. Individuals with CRD were associated with a significantly lower rate of lung and bronchus cancer (0.16% vs 0.31%; RR 0.53; 95% CI, 0.48–0.59; *P* < .001) and reactive airway disease; asthma (6.30% vs 7.37%; RR 0.86; 95% CI, 0.84–0.87; *P* < .001) and COPD (1.65% vs 3.05%; RR 0.54; 95% CI, 0.52–0.56; *P* < .001) than those with cigarette smoking.

**Conclusion:**

We found that cannabis use was associated with higher risks of mortality, stroke, myocardial infarction, and suicide for ED patients when compared to cigarette smoking. Cannabis-related disorder was associated with lower rates of reactive airway disease and lung and bronchus cancer when compared to those with cigarette smoking.

## INTRODUCTION

The relationship between tobacco smoking and adverse health outcomes has been extensively studied and well-documented over the past several decades. Numerous studies have established strong associations between tobacco use and cardiovascular disease, acute and chronic respiratory illness, and an increased risk of cancer, among other conditions. 2 With growing public awareness and robust public health campaigns highlighting the dangers of tobacco use, smoking rates in the U.S. have steadily declined. In 2022, the smoking rate among adults in the U.S. was 11.6%, a substantial decrease from 42.6% reported in 1965. 3 Even between 2006 and 2019, a notable decline in smoking prevalence was observed, but this may be due to the transition from traditional cigarettes to vaping as a nicotine delivery device. 4

In contrast, cannabis use has increased over the past decade. This rise in use has been driven in part by the growing legalization of cannabis for both medicinal and recreational purposes. Among adults 19 to 30 years of age, daily cannabis use nearly doubled from 6% in 2012 to 11% in 2022. 5 As more states legalize cannabis, usage rates are expected to continue climbing. Although the health effects of cannabis are less well-characterized than those of tobacco, emerging research has identified a potential association between cannabis use and elevated risks of cardiovascular events, particularly myocardial infarction and stroke. 6–8

Despite these associations, cannabis is “consistently perceived as more socially acceptable and less risky than cigarettes” by the general public. 9 This perception may not be entirely accurate, and further research is needed to better understand and compare the health outcomes of cannabis and tobacco use. However, much of the existing literature evaluates cannabis outcomes while adjusting for tobacco exposure or excluding tobacco users, rather than directly contrasting cannabis and cigarette smoking within the same analytic framework. As a result, few studies provide true head-to-head comparisons of cannabis use versus cigarettes to better contextualize relative risk and inform public health messaging.^10^,^11^

Cannabis use and cigarette smoking are both common among emergency department (ED) patients and frequently encountered in acute care settings. Given that the ED often serves patients with substance use disorders and other high-risk conditions, characterizing the risks associated with these exposures may help identify higher-risk patients, inform counseling strategies, and support preventive interventions.

### Objectives of Investigation

Given the increasing prevalence of cannabis use, this study aims to compare the health effects of cannabis and cigarette use by evaluating outcomes including all-cause mortality, stroke, myocardial infarction, suicide, lung and bronchus cancer, and reactive airway disease among ED patients.

**Population Health Research Capsule**

What do we already know about this issue?

*Cannabis use is increasing and often perceived as safer than cigarettes, but direct comparisons of health outcomes are limited.*

What was the research question?

*Do emergency department patients who use cannabis have different health outcomes than cigarette smokers?*

What was the major finding of the study?

*Cannabis was associated with increased mortality (RR 1.09; 95% CI, 1.05–1.12; P < .001) and also suicide risk vs cigarettes (RR 1.77; 95% CI, 1.65–1.90; P < .001).*

How does this improve population health?

*These results contribute to improved patient counseling and public health messaging by clarifying the relative risks of cannabis versus cigarette use.*

## METHODS

This retrospective, propensity score–matched study used the TriNetX database, an international, federated health research network providing access to de-identified electronic health records (EHR) across healthcare organizations, to analyze the records of 120,689,106 patients from 69 healthcare organizations in the U.S. between 2005 and 2022. The TriNetX database includes both academic medical centers and affiliated community healthcare facilities, providing broad representation of US healthcare.

### Cohort Selection

The first cohort consisted of adult patients (≥18 years of age at the time of diagnosis) and was grouped based on a diagnosis of cannabis-related disorders (CRD; *International Classification of Diseases, 10^th^ Revision, Clinical Modification* [*ICD-10-CM*] code F12) within five years before or one month after an ED visit. The second cohort consisted of adult patients diagnosed with “nicotine dependence, cigarettes” (ICD-10-CM: F17.21) within five years before or one month after an ED visit [Supplementary Table 1]. A five-year preindex and one month postindex exposure window was selected to capture chronic substance use while accounting for delayed documentation of preexisting CRD and nicotine dependence. Patients were excluded from the study if they were <18 years of age or the ED visit occurred before January 2005 or after January 2022. Additionally, patients who previously experienced the outcomes or belonged to both cohorts were excluded from the study. We selected ED visits to make both groups comparable and allow propensity score matching. The TriNetX database limits propensity matching in very large cohorts. Two cohorts greater than five million patients each results in increased instability in the TriNetX network and limits the number of covariates used for propensity matching. When there are two cohorts greater than 10 million patients each, the propensity matching generally fails.

TriNetX does not impute or estimate clinical values in patient records, except in specific cases not applicable to this study. If patient encounters are missing or have inconsistent encounter IDs, TriNetX derives an encounter using the earliest and latest observation dates but does not assign an encounter type. For encounters lacking start or end dates, the platform uses associated observation dates to fill in the gaps. Transition from *ICD-9* to *ICD-10* were done internally by TriNetX within the database. We did not perform data-cleaning, deduplication, filtering, or preprocessing.

### Outcomes Measured

The primary outcomes were assessed within three years after the initial ED visit and included all-cause mortality, stroke, myocardial infarction, suicide, cancer of the lung and bronchus, and reactive airway disease (asthma and chronic obstructive pulmonary diseases [COPD]). These outcomes were defined with their corresponding *ICD-10* codes as follows: all-cause mortality; stroke (*ICD-10-CM*: I63) myocardial infarction (*ICD-10-CM*: I21), suicide *(ICD-10-CM*: T14.91), cancer of the lung and bronchus (*ICD-10-CM*: C34); or reactive airway disease (asthma *ICD-10-CM*: J45 and COPD *ICD-10-CM*: J44) [Supplementary Table 1]. Measuring outcomes three years out allowed us to associate early onset of both long-term outcomes such as lung cancer, and short-term outcomes such as myocardial infarction. The index date was the ED visit.

Patients were observed for outcomes in TriNetX until the earliest of outcome occurrence, three years after the index date, the last recorded encounter within the network, or the end of site data availability. Nonfatal outcomes were identified from encounter diagnoses within participating healthcare organizations. All-cause mortality was identified through the TriNetX platform. Mortality information in TriNetX is obtained from death indicators submitted by participating healthcare organizations and may be supplemented by external mortality data sources, including national death registry data for participating organizations with such linkage. Because linkage availability may vary across healthcare organizations and over time, mortality ascertainment may be incomplete for patients whose deaths were not captured in the EHR or linked mortality sources. Patients were further excluded if they had outcomes prior to the initial ED visit [Supplementary Table 2].

### Statistical Analysis

We performed a 1:1 propensity score–matching approach using greedy nearest-neighbor matching with a caliper width threshold of 0.10 (or 10%). Propensity matching was performed with linear regression for continuous variables such as age and logistic regression for binary (yes/no) variables. We matched patients for demographics and the seven preexisting conditions associated with mortality [Table 1]. The comorbidities were chosen based on the leading causes of death according to the Centers for Disease Control and Prevention analysis within the TriNetX platform for each cohort and reported as descriptive statistics for the measured outcomes with the following: risk ratios (RR), 95% CIs, and probability values (*P* values). Risk ratios were reported rather than odds ratios (ORs) as RRs are more intuitive to the average practicing physician.

The use of de-identified data from TriNetX has been determined to be exempt by the University of Texas Medical Branch (UTMB) Institutional Review Board (IRB). The UTMB IRB determined that this project did not involve intervention or interaction with human subjects, and is de-identified per the de-identification standard defined in Section §164.514(a) of the Health Insurance Portability and Accountability Act Privacy Rule. This formal determination by a qualified expert refreshed on December 2020. This retrospective cohort study was conducted and reported in accordance with the Strengthening the Reporting of Observational Studies in Epidemiology guidelines.^13^

## RESULTS

### Cohort Characteristics

A total of 1,682,117 patients were identified with ED visits and either CRD or cigarette smoking. After propensity matching, there were a total of 651,232 adult patients with CRD (325,616) or cigarette smoking (325,616) within five years prior or one month after an ED visit (Figure). There were 2,713 patients excluded because they met the index event more than 20 years ago.

Propensity matching substantially improved age balance between groups, reducing the mean age difference from 33.3 versus 43.8 years before matching to 33.8 versus 34.1 years after matching (standardized difference = 0.02). Before propensity matching, the male:female ratio was 1.43:1 in the CRD cohort and 1.07:1 in the cigarette smoking cohort, which narrowed after propensity matching. After propensity matching, some of the covariates remained statistically different; however, all the standard mean differences of the covariates were < 0.1, representing a well balanced match (Table 1).

### Main Results

Before propensity matching (Table 2), the CRD cohort, which was significantly younger, was associated with a lower risk of all-cause mortality compared to cigarette smoking (2.30% vs 3.92%; RR, 0.59; 95% CI, 0.57–0.60; *P* < .001). Similarly, CRD was associated with lower risks of stroke (0.43% vs 0.54%; RR, 0.80; 95% CI, 0.75–0.84; *P* < .001), myocardial infarction (0.52% vs. 0.67%; RR, 0.78; 95% CI, 0.74–0.82; *P* < .001), and COPD (1.60% vs 6.86%; RR, 0.23; 95% CI, 0.23–0.24; *P* < .001). In contrast, tetrahydrocannabinol (THC) use was associated with a higher risk of suicide compared to cigarette smoking (0.62% vs 0.31%; RR, 2.03; 95% CI, 1.92–2.14; *P* < .001). Lung cancer (0.16% vs 0.82%; RR, 0.19; 95% CI, 0.18–0.21; *P <* .001) and asthma (6.36% vs. 6.69%; RR, 0.95; 95% CI, 0.94–0.97; *P* < .001) were also less prevalent in CRD patients before matching.

After propensity matching [Table 2], CRD was associated with a higher rate of all-cause mortality compared to cigarette smoking (2.35% vs 2.16%; RR, 1.09; 95% CI, 1.05–1.12; *P* < .001). Similarly, CRD was associated with an increased risk of stroke (0.45% vs 0.28%; RR, 1.59; 95% CI, 1.47–1.73; *P* < .001) and myocardial infarction (0.53% vs. 0.35%; RR, 1.51; 95% CI, 1.40–1.63; *P* < .001). The use of THC was also associated with a higher risk of suicide compared to cigarette smoking (0.62% vs 0.35%; RR, 1.77; 95% CI, 1.65–1.90; *P* < .001). In contrast, CRD was associated with a lower risk of lung and bronchus cancer (0.16% vs 0.31%; RR, 0.53; 95% CI, 0.48–0.59; *P* < .001), asthma (6.30% vs.7.37%; RR, 0.86; 95% CI, 0.84–0.87; *P* < .001), and COPD (1.65% vs 3.05%; RR, 0.54; 95% CI, 0.52–0.56; *P* < .001).

Before matching, THC use appeared to be associated with a lower risk of mortality, stroke, myocardial infarction, and COPD compared to cigarette smoking, while suicide rates were higher among THC users. However, after matching for demographics and preexisting conditions, the association reversed, with THC use demonstrating a higher risk of mortality, stroke, myocardial infarction, and suicide, likely related to the 10-year age gap that disappeared after propensity matching. The increased prevalence of lung cancer, asthma, and COPD in cigarette-smoking individuals remained consistent before and after matching (Table 2).

A post hoc sample-size assessment was performed for comparison of two independent proportions using a two-sided α of .05 and 80% power. Based on the observed post–propensity-matched event rates, the most conservative outcome was all-cause mortality, with rates of 2.35% in the CRD cohort and 2.16% in the cigarette-smoking cohort. Detecting this absolute difference of 0.19% required approximately 95,845 patients per group. The required sample sizes for the remaining outcomes were lower, ranging from approximately 1,837 patients per group for COPD to 21,223 patients per group for myocardial infarction. Because the final propensity-matched analysis included 325,616 patients in each cohort, the available sample size exceeded the calculated requirement for all outcomes, indicating adequate statistical power to detect the observed between-group differences.

## DISCUSSION

The objective of this study was to compare rates of various medical conditions between cannabis and cigarette users who visited the ED. This study demonstrated that cannabis use was associated with higher risks of mortality, stroke, myocardial infarctions, and suicide for ED patients when compared to cigarette use. Conversely, cigarette smoking was associated with higher rates of reactive airway disease and lung and bronchus cancer relative to cannabis use. Notably, direct head-to-head comparisons between cannabis use and cigarette smoking remain uncommon in the contemporary literature, as tobacco is frequently treated as a confounder or an exclusion criterion in cannabis-outcome studies. By evaluating cannabis and cigarette users side by side using the same data source and analytic framework, our findings add comparative evidence that may better inform risk perception and patient counseling.^10^,^11^

Cannabis is often perceived as safer than cigarette smoking, with many individuals believing that daily cannabis use is less harmful than daily cigarette use.^14^ This misconception can be dangerous, contributing to increased cannabis consumption despite mounting evidence of adverse health outcomes. This study highlights associations between cannabis use and cardiovascular disease, as well as suicidal behavior, suggesting that cannabis may not be as benign as it is commonly perceived. Cannabis is the most commonly used illicit drug in the U.S. and is the second leading cause of substance-related ED visits.^15^,^16^ The psychoactive compound in cannabis, THC, exerts systemic cardiovascular and neurological effects, including vasoconstriction, enhanced sympathetic and parasympathetic activity, atherosclerosis, and platelet aggregation.^17^ Prior studies have shown correlations between cannabis use and increased risks of cardiac arrhythmias, myocardial infarction, and stroke. 6–8

In addition, cannabis use is associated with mood disturbances, including increased suicidal ideation, planning, and attempts. Public awareness of these negative consequences remains limited, especially in contrast to the well-documented risks of cigarette smoking, making cannabis a deceptively dangerous substance. Over the past 40 to 50 years, cannabis potency has increased from 2% to 3% in the 1970s to 15% to 30% currently and up to 90% in concentrates.^18^–^21^ This increase in potency is due to selective breeding, improvements in indoor cultivation technology, legalization driving competition for stronger products, and a shift from smoking the marijuana flower to concentrates with much higher THC exposure.^18^–^22^ Previous studies have shown increased ED visits among cannabis users for cardiovascular and psychological complaints.

Cannabis use has been associated with several acute and chronic cardiovascular effects, largely attributed to its primary psychoactive component, THC. Tetrahydrocannabinol activates the sympathetic nervous system, leading to increased heart rate and blood pressure through catecholamine release, thereby raising myocardial oxygen demand.^23^,^24^ This heightened demand, compounded by reduced coronary perfusion due to elevated carboxyhemoglobin levels from smoking, creates an oxygen supply-demand mismatch that can precipitate ischemia and infarction—particularly in individuals with preexisting cardiovascular disease.^23^,^25^ Population-level data and case reports have linked cannabis use to arrhythmias, myocardial infarction, and stroke, with risk of myocardial infarction onset increased nearly fivefold in the hour after use.^23^, ^25^–^27^

In addition, THC may promote endothelial dysfunction and platelet aggregation through cannabinoid receptor type 1 (CB1) and type 2 (CB2) activation, contributing to a prothrombotic state.^23^,^24^ Experimental and clinical data suggest that cannabis exposure can impair vascular endothelial function to a magnitude comparable to tobacco smoke. In a rat model, 1 minute of secondhand cannabis smoke exposure substantially impaired endothelial function for at least 90 minutes, lasting longer than comparable impairment from tobacco secondhand smoke exposure.^28^ Furthermore, in a cross-sectional human study, chronic cannabis smoking and THC-edible use were associated with endothelial dysfunction similar to tobacco smokers, albeit apparently occurring via distinct mechanisms.^29^ Tetrahydrocannabinol likely mediates many acute cardiovascular effects of cannabis; however, overall cardiovascular risk is also influenced by smoke exposure, route of administration, product potency, and co-use of other substances.

Beyond cardiovascular risk, growing evidence suggests a strong association between cannabis use and suicidal ideation, attempts, and death by suicide—particularly in adolescents and young adults.^30^ Tetrahydrocannabinol acts on CB1 receptors located in brain regions critical to emotion regulation, including the prefrontal cortex, amygdala, and hippocampus. Chronic exposure may lead to downregulation of these receptors, disruption of endocannabinoid tone, and impairments in serotonergic and dopaminergic neurotransmission–neurobiological changes commonly implicated in depression and impulsivity.^31^,^32^ A large meta-analysis found cannabis use to be significantly associated with increased odds of suicidal ideation (OR 1.50) and suicide attempts (OR 3.46).^31^ Additionally, a large real-world, retrospective study demonstrated a 7.80-fold higher risk of suicide attempts in those with cannabis use disorder (CUD) compared to those in a control population without CUD.^30^ Neuroimaging and behavioral studies further support these findings, showing cannabis-related deficits in executive function and increased impulsivity, which may heighten vulnerability to self-harm.^31^,^32^ These psychiatric effects are further amplified by the high prevalence of comorbid mental health conditions among cannabis users.

These results underscore the consistency of evidence across multiple studies illustrating the adverse health effects of cannabis. The widespread prevalence of cannabis use may partly stem from the relative absence of public health campaigns targeting cannabis-related harms. Raising public awareness of its cardiovascular and psychiatric risks could help reduce ED visits and promote better population health. The data presented here can contribute to broader educational efforts aimed at reducing cannabis use and informing the public about its potential health consequences.

By contrast, this study reaffirms the strong association between cigarette smoking and respiratory diseases, including reactive airway disease and lung and bronchus cancers. There is substantial evidence linking cigarette use to COPD, pulmonary fibrosis, atherosclerosis, coronary artery disease, sexual dysfunction, and numerous other conditions. 11 The pathophysiological effects of tobacco smoke and its carcinogenic properties have been extensively documented, establishing a clear causal relationship between cigarette use and pulmonary malignancies. Unlike cannabis, cigarette smoking has long been the focus of public health interventions, with a substantial shift in public perception recognizing its dangers.^33^ As a result, tobacco use has declined steadily over the past five decades. Nevertheless, approximately 11.5% of the U.S. population continues to use tobacco products, representing a significant public health burden.^32^ The present findings further support existing literature by reinforcing the link between cigarette use and adverse respiratory and oncologic outcomes.

## LIMITATIONS

This study had several limitations, primarily owing to its retrospective design. While correlations can be observed, the retrospective nature limits the ability to control for all unmeasured confounding variables and selection biases, allowing only for the reporting of associations between cohorts and outcomes. Although this database methodology is valuable for generating hypotheses, future prospective studies are needed to establish causal relationships.

Additionally, because the study relied on TriNetX data, the accuracy of. data entry could not be confirmed. Furthermore, TriNetX privacy policies prevented the assessment of region-specific data points, which may have introduced selection bias. Finally, outcome events —including death —may have been missed if patients received care at a healthcare organization outside the TriNetX network. This limitation is mitigated for mortality outcomes, as approximately 94% of participating healthcare organizations are currently linked to US death registries, a proportion that continues to increase over time. However, nonfatal outcomes such as myocardial infarction, suicide attempt, stroke, lung cancer, or reactive airway disease may be underascertained if follow-up occurs outside the TriNetX network.

This study population was derived from patients presenting to the ED, which may not be representative of the general adult population, as these individuals are more likely to have higher acuity illness and comorbidity burden; therefore, the findings may have limited generalizability to broader community-based populations. In addition, the three-year observation period may underestimate chronic exposure outcomes with long-latency periods. The possibility of exposure misclassification, residual confounding, selection bias, competing-risk bias, survivor bias, and immortal time bias may also compromise interpretation of the reported RRs, particularly for outcomes such as lung cancer and mortality.

Differential healthcare usage patterns between cohorts may have further compounded this issue. Prior to matching, the cigarette-smoking cohort was substantially older and likely carried a greater burden of chronic comorbidity, potentially resulting in more frequent healthcare encounters and greater retention within the healthcare network compared with younger cannabis-using patients. Patients lost to follow-up after only several months without experiencing an event were analytically treated the same as patients observed continuously for the full 3-year period. This limitation introduces important censoring, survivor, and competing-risk biases that may not be adequately addressed in the current statistical framework.

Another limitation can be attributed to incorrect patient responses. Cohorts were created based on *ICD-10* codes for CRD and nicotine dependence, cigarettes. The transition from *ICD-9* to *ICD-10* relies on administrative coding, which may represent another limitation of this study. Patients may underreport THC or cigarette use due to fear of judgment or incomplete disclosure of their substance use history, and those who use these substances may also use other recreational drugs influencing health outcomes. Additionally, cigarette smoking was defined using diagnoses of nicotine dependence, although only approximately 80% of daily smokers meet criteria for nicotine dependence; however, these terms were used interchangeably. 1,3

Among daily or near-daily cannabis users, approximately 30% meet criteria for CRD, and CRD encompasses a broad spectrum including cannabis use, abuse, and dependence. Consequently, this analysis likely reflects outcomes among heavier or more frequent users of THC than tobacco, rather than occasional users. Furthermore, the database lacked information to differentiate between smoking and oral intake of drugs or to identify patients using multiple routes of administration; however, exclusive oral users represent a small minority (<10%).^34^ Although unreported or subclinical cannabis use could not be captured, this study primarily represents individuals with CUD who are more likely to experience related complications. Accordingly, the findings may not be generalizable to individuals with lower levels of substance exposure.

While this study propensity matched for cigarette and THC use, age, sex, race, and preexisting conditions associated with mortality, residual confounding may persist because not all potential confounding variables were available within the database coding system. Other factors, such as a patient’s socioeconomic status, healthcare utilization, use of other recreational substances, medications, psychiatric illnesses, and diet may all contribute to outcomes. The frequency or duration of use, route of administration, cumulative exposure for tobacco (pack-years) or cannabis (potency), or concurrent polysubstance use could not be controlled for, and this represents a limitation. Additionally, temporal increases in cannabis potency may influence outcomes and warrant further investigation in longitudinal analyses.^18^–^22^ Future prospective studies could provide additional information on potential contributing factors such as mechanisms, polysubstance use, or socioeconomic factors.

## CONCLUSION

This study highlights important differences in the health outcomes associated with cannabis and cigarette use among ED patients. Cannabis use was associated with higher rates of mortality, stroke, myocardial infarction, and suicide, whereas cigarette smoking—defined by nicotine dependence—was more strongly associated with reactive airway disease and lung and bronchus cancer.

These findings are relevant to public health, particularly given increasing cannabis potency and the perception that cannabis is relatively safe. Understanding these different risks associated with cannabis and cigarette use may help guide patient counseling, public messaging, and health education efforts.

## Supplementary Information



## Figures and Tables

**Figure:**
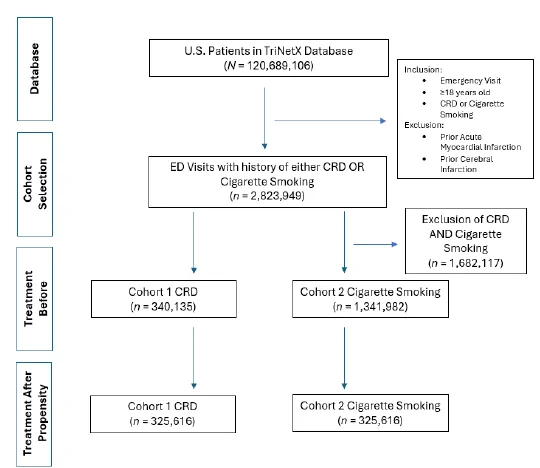
Flow chart of participant cohort selection in a study of the relationship between cannabis use and cigarette smoking on mortality, cardiovascular outcomes, suicide, cancer of the lung and bronchus, and reactive airway disease in emergency department patients. *CRD*, cannabis-related disorders; *ED*, emergency department; *US*, United States.

**Table 1:** Demographics and preexisting conditions of emergency department patients using tetrahydrocannabinol vs cigarette smokers before and after propensity score matching.

	Before Propensity Score Matching					After Propensity Score Matching					
Demographics											
Cohort 1 = THC 2 = Cig		Mean (SD)	Patients	% of Cohort	*P* value	Std diff.	Mean (SD)	Patients	% of Cohort	*P* value	Std diff.
1	Age at Index	33.3 (13.6)	337,449	100%	< .001	0.723	33.8 (13.6)	325,616x	100%	< .001	0.020
2		43.8 (15.3)	1,341,955	100%			34.1 (13.4)	34.1 (13.4)	100%		
1	Female		136,002	40.3%	< .001	0.111		133,767	41.1%	< .001	0.041
2			614,428	45.8%				127,168	39.1%		
1	Black or African American		102,930	30.5%	< .001	0.290		93,128	28.6%	< .001	0.012
2			243,915	18.2%				94,916	29.1%		
1	Male		194,613	57.7%	< .001	0.172		185,015	56.8%	< .001	0.042
2			659,463	49.1%				191,800	58.9%		
1	White		166,304	49.3%	< .001	0.259		166,049	51.0%	< .001	0.018
2			832,489	62.0%				163,124	50.1%		
1	American Indian or Alaska Native		1,226	0.4%	= .013	0.005		1,217	0.4%	= .87	< .001
2			4,503	0.3%				1,209	0.4%		
1	Unknown Race		45,671	13.5%	< .001	0.009		44,006	13.5%	< .001	0.014
2			177,327	13.2%				45,605	14.0%		
1	Native Hawaiian or Other Pacific Islander		2,921	0.9%	< .001	0.017		2,919	0.9%	= .003	0.007
2			13,771	1.0%				2,696	0.8%		
1	Unknown Gender		6,834	2.0%	< .001	0.165		6,834	2.1%	= .11	0.004
2			68,064	5.1%				6,648	2.0%		
1	Hispanic or Latino		31,856	9.4%	< .001	0.119		30,109	9.2%	= .13	0.004
2			83,815	6.2%				30,467	9.4%		
1	Not Hispanic or Latino		204,328	60.6%	< .001	0.032		196,885	60.5%	< .001	0.009
2			833,446	62.1%				195,424	60.0%		
1	Other Race		12,779	3.8%	< .001	0.031		12,682	3.9%	= .42	0.002
2			43,184	3.2%				12,809	3.9%		
1	Asian		5,618	1.7%	< .001	0.025		5,615	1.7%	< .001	0.009
2			26,766	2.0%				5,257	1.6%		
Diagnosis											
Cohort			Patients	% of Cohort	*P* value	Std diff.		Patients	% of Cohort	*P* value	Std diff.
1	Ischemic heart diseases		8,695	2.6%	< .001	0.159		8,623	2.6%	= .75	0.001
2			77,071	5.7%				8,664	2.7%		
1	Diabetes mellitus		18,792	5.6%	< .001	0.157		18,568	5.7%	= .51	0.002
2			130,743	9.7%				18,691	5.7%		
1	Overweight and obesity		30,418	9.0%	< .001	0.075		29,705	9.1%	= .04	0.005
2			151,538	11.3%				29,223	9.0%		
1	Acute kidney failure and chronic kidney disease		14,106	4.2%	< .001	0.052		13,457	4.1%	< .001	0.012
2			70,919	5.3%				14,260	4.4%		
1	Heart failure		5,783	1.7%	< .001	0.087		5,686	1.7%	= .30	0.003
2			40,719	3.0%				5,576	1.7%		
1	Cardiac arrest		396	0.1%	= .82	< .001		384	0.1%	< .001	0.012
2			1,555	0.1%				264	0.1%		
1	Hypertensive diseases		45,586	13.5%	< .001	0.247		44,927	13.8%	< .001	0.009
2			308,599	23.0%				45,987	14.1%		

*Cig*, cigarette smoking; *SD*, standard deviation; *Std diff*, standard difference; *THC*, tetrahydrocannabinol.

**Table 2:** Comprehensive outcomes of emergency department patients using tetrahydrocannabinol vs cigarette smoking before and after propensity score matching.

	Before Propensity Score Matching					After Propensity Score Matching				

Outcomes	THC (%)	Nicotine (%)	RR (95% CI)	OR (95% CI)	*P* Value	THC (%)	Nicotine (%)	RR (95% CI)	OR (95% CI)	*P* Value
Deceased	7,752 (2.30%)	52,519 (3.92%)	0.59 (0.57–0.60)	0.58 (0.56–0.59)	< .001	7,644 (2.35%)	7,029 (2.16%)	1.09 (1.05–1.12)	1.09 (1.05,1.13)	< .001
Stroke	1,459 (0.43%)	7,300 (0.54%)	0.80 (0.75–0.84)	0.79 (0.75–0.84)	< .001	1,454 (0.45%)	913 (0.28%)	1.59 (1.47–1.73)	1.60 (1.47, 1.73)	< .001
MI	1,755 (0.52%)	8,922 (0.67%)	0.78 (0.74–0.82)	0.78 (0.74–0.82)	< .001	1,738 (0.53%)	1,152 (0.35%)	1.51 (1.40–1.63)	1.51 (1.40, 1.63)	< .001
Lung Cancer	533 (0.16%)	10,913 (0.82%)	0.19 (0.18–0.21)	0.19 (0.18–0.21)	< .001	533 (0.16%)	1,000 (0.31%)	0.53 (0.48–0.59)	0.53 (0.48, 0.59)	< .001
Suicide	2,090 (0.62%)	4,111 (0.31%)	2.03 (1.92–2.14)	2.03 (1.93–2.14)	< .001	2,017 (0.62%)	1,142 (0.35%)	1.77 (1.65–1.90)	1.78 (1.65, 1.91)	< .001
Asthma	19,290 (6.36%)	81,607 (6.69%)	0.95 (0.94–0.97)	0.95 (0.93–0.96)	< .001	18,454 (6.30%)	21,818 (7.37%)	0.86 (0.84–0.87)	0.85 (0.83, 0.86)	< .001
COPD	5,274 (1.60%)	84,128 (6.86%)	0.23 (0.23–0.24)	0.22 (0.21–0.23)	< .001	5,247 (1.65%)	9,579 (3.05%)	0.54 (0.52–0.56)	0.53 (0.52, 0.55)	< .001

*COPD*, chronic obstructive pulmonary disease; *MI*, myocardial infarction; *OR*, odds ratio; *RR*, relative risk; *THC*, tetrahydrocannabinol.
