# Supplementary material for: Cannabis Use versus Cigarette Smoking in Emergency Department Patients: Comparative Outcomes
Source: West J Emerg Med. 2026 Oct 14;27(6):1735–43. doi: 10.5811/westjem.64353 (PMC13762010; doi:10.5811/westjem.64353)
Supplement: Supplementary file 1 [file wjem-27-1735-s001.docx]

| **Supplemental Table 1: Codes used in study.** | |  |
| --- | --- | --- |
| **Demographics** | **Category** | **Code** |
| American Indian or Alaska Native | Demographics | 1002-5 |
| Asian | Demographics | 2028-9 |
| Black or African American | Demographics | 2054-5 |
| Native Hawaiian or Other Pacific Islander | Demographics | 2076-8 |
| White | Demographics | 2106-3 |
| Other Race | Demographics | 2131-1 |
| Hispanic or Latino | Demographics | 2135-2 |
| Not Hispanic or Latino | Demographics | 2186-5 |
| Age at Index | Demographics | AI |
| Female | Demographics | F |
| Male | Demographics | M |
| Unknown Gender | Demographics | UN |
| Unknown Race | Demographics | UNK |
| **Diagnosis** |  |  |
| Malignant neoplasm of bronchus and lung | ICD-10-CM | C34 |
| Diabetes mellitus | ICD-10-CM | E08-E13 |
| Overweight and obesity | ICD-10-CM | E66 |
| Alcohol related disorders | ICD-10-CM | F10 |
| Cannabis related disorders | ICD-10-CM | F12 |
| Cocaine related disorders | ICD-10-CM | F14 |
| Nicotine dependence, cigarettes | ICD-10-CM | F17.21 |
| Hypertensive diseases | ICD-10-CM | I10-I1A |
| Ischemic heart diseases | ICD-10-CM | I20-I25 |
| Acute myocardial infarction | ICD-10-CM | I21 |
| Cardiac arrest | ICD-10-CM | I46 |
| Heart failure | ICD-10-CM | I50 |
| Cerebral infarction | ICD-10-CM | I63 |
| Other chronic obstructive pulmonary disease | ICD-10-CM | J44 |
| Asthma | ICD-10-CM | J45 |
| Acute kidney failure and chronic kidney disease | ICD-10-CM | N17-N19 |
| Suicide attempt | ICD-10-CM | T14.91 |
| **Visit** |  |  |
| Emergency | HL7V3.0 | VisitType:EMERGENCY |
| **Mortality** |  |  |
| Deceased | Demographics | Deceased |

| Supplementary Table 2 : Patients excluded from results because they had the outcome prior to the time window. | | | | | | | |
| --- | --- | --- | --- | --- | --- | --- | --- |
|  | | Non-Propensity | | | Propensity | | |
| 1 = THC  2 = Cig | Outcome | Patients in Cohort | Patients with Outcome | Excluded | Patients in Cohort | Patients with Outcome | Excluded |
| 1  2 | Death | 337,158  1,340,050 | 7,752  52,519 | 291  1,905 | 325,332  325,305 | 7,644  7,029 | 284  311 |
| 1  2 | Stroke | 337,246  1,341,020 | 1,459  7,300 | 203  935 | 325,416  325,510 | 1,454  913 | 200  106 |
| 1  2 | Myocardial infarction | 337,255  1,340,951 | 1,755  8,922 | 194  1,004 | 325,423  325,502 | 1,738  1,152 | 193  114 |
| 1  2 | Lung cancer | 336,596  1,330,662 | 533  10,913 | 853  11,293 | 324,764  324,614 | 533  1,000 | 852  1,002 |
| 1  2 | Suicide | 335,845  1,339,134 | 2,090  4,111 | 1,604  2,821 | 324,065  324,856 | 2,017  1,142 | 1,551  760 |
| 1  2 | Asthma | 303,225  1,219,190 | 19,290  81,607 | 34,224  122,765 | 293,022  296,059 | 18,454  21,818 | 32,594  29,557 |
| 1  2 | COPD | 330,126  1,226,448 | 5,274  84,128 | 7,323  115,507 | 318,319  313,970 | 5,247  9,579 | 7,297  11,646 |
| Cig = cigarette smoking; COPD = chronic obstructive pulmonary disease; THC = tetrahydrocannabinol | | | | | | | |

**Greedy Nearest-Neighbor Matching**

The most common implementation of propensity matching is pair-matching, in which pairs of treated and control participants are formed. There are several common implementations of pair-matching. The most commonly used is greedy nearest-neighbor matching (NNM), which we used, in which a treated participant is selected at random and then matched to the control participant whose propensity score is closest to that of the treated participant. The process is described as greedy because at each stage the control is selected who is closest to the currently considered treated participant, even if that untreated participant would serve better as a control for a subsequent treated participant. This process is then repeated until a matched control participant has been selected for each treated participant. This process generally uses matching without replacement, so that once a control participant is matched to a treated participant, that control participant is no longer available for matching to a subsequent treated participant. A reﬁnement is nearest-neighbor matching with a caliper restriction. Using this approach, a control participant is an acceptable match for a treated participant only if the diﬀerence in their propensity scores is less than a maximum amount (the caliper width or distance). For technical reasons, one typically matches on the logit of the propensity score and uses a caliper width that is deﬁned as a proportion (0.1-0.2) of the standard deviation (SD) of the logit of the propensity score. A crucial step in any study that uses propensity matching is to assess the degree to which matching on the propensity score resulted in the formation of a matched sample in which the distribution of baseline characteristics is similar between treated and control participants. This assessment is critical as it allows both the researcher and readers to assess whether matching on the estimated propensity score has removed systematic baseline diﬀerences between treatment. The use of the standardized diﬀerence, which is the diﬀerence in means in units of SD, is often used for assessing the similarity of matched treated and control participants. Some authors have suggested that a threshold of 0.10 (or 10%) be used to denote acceptable balance after matching. Once acceptable balance has been achieved, analysts can unblind themselves to the outcome and compare outcomes between treated and control participants in the matched sample. The analyses conducted in the propensity score–matched sample can be similar to those that would be done in a randomized controlled trial with a similar outcome.
